# Association between Serum Vitamin D Levels and Sleep Disturbance in Hemodialysis Patients

**DOI:** 10.3390/nu9020139

**Published:** 2017-02-14

**Authors:** Bin Han, Fu-Xiang Zhu, Chao Shi, Heng-Lan Wu, Xiao-Hong Gu

**Affiliations:** Department of nephrology, First Affiliated Hospital of Jiaxing University, Jiaxing 314000, China; zfxywh@163.com (F.-X.Z.); shichao@ymail.com (C.S.); wh1525487@sina.com (H.-L.W.); zjl1499525408@sina.com (X.-H.G.)

**Keywords:** vitamin, sleep disturbance, hemodialysis

## Abstract

Sleep disturbance is a frequent and serious complication of hemodialysis (HD). Low serum vitamin D levels have been associated with sleep quality in non-HD subjects. Our aim was to examine the possible association between serum vitamin D levels and the presence of sleep disturbance in HD patients. We recruited 141 HD patients at the HD center of the First Affiliated Hospital of Jiaxing University during 2014–2015. Serum levels of 25-hydroxyvitamin D (25(OH)D) were determined by the competitive protein-binding assay. Sleep quality was measured using the Pittsburgh Sleep Quality Index (PSQI). Demographic, clinical and laboratory data were recorded. Meanwhile, 117 healthy control subjects were also recruited and underwent measurement of 25(OH)D. Eighty-eight patients (62.4%) had sleep disturbance (PSQI scores ≥ 5). Patients with sleep disturbance showed lower levels of 25(OH)D as compared to those without sleep disturbance (85.6 ± 37.4 vs. 39.1 ± 29.1 nmol/L, *p* < 0.001). In multivariate analyses, serum levels of 25(OH)D (≤48.0 nmol/L) were independently associated with sleep disturbance in HD patients (OR 9.897, 95% CI 3.356–29.187, *p* < 0.001) after adjustment for possible variables. Our study demonstrates that low serum levels of vitamin D are independently associated with sleep disturbance in HD patients, but the finding needs to be confirmed in future experimental and clinical studies.

## 1. Introduction

Sleep disturbance is extremely common in hemodialysis (HD) patients, with prevalence ranging from 41% to 83% [1,2,3,4]. The presence of sleep disturbance has been associated with reduced quality of life [3,5] and increased mortality [3,6] in HD patients. Moreover, it has been reported to be involved in the development of cardiovascular diseases in patients undergoing maintenance HD [7]. However, the pathogenesis of sleep disturbance in HD patients remains unclear.

As a fundamental micronutrient, vitamin D is extremely essential for human health [8]. A large body of preclinical studies has found the profusion of vitamin D receptors in specific areas of the brainstem that are thought to regulate sleep [9,10,11]. Furthermore, increasing clinical studies have shown that low serum levels of vitamin D are associated with poorer sleep, including low sleep efficiency and short sleep duration, in non-HD subjects, suggesting a potential role for vitamin D in maintaining healthy sleep [12,13]. Similarly, a significantly correlation between vitamin D levels and sleep quality has been found in patients with systemic lupus erythematosus (SLE) [14]. Recent uncontrolled clinical trials of vitamin D supplements in patients with sleep problems have reported improved sleep quality with higher levels of supplemental vitamin D [15,16].

At the global level, about one billion people have vitamin D insufficiency/deficiency [8]. In patients with chronic kidney disease (CKD) stage 5 on HD, the prevalence of vitamin D insufficiency/deficiency is up to 96.6% [17]. The presence of vitamin D anomalies has been associated with increased risk of cardiovascular outcomes and mortality in patients with end-stage renal disease (ESRD) on HD [18,19]. To date, however, no study has evaluated the potential relationship of vitamin D to sleep disturbance in HD patients. Given the involvement of vitamin D in sleep quality in non-HD subjects and the acknowledged high prevalence of vitamin D anomalies in HD patients, whether serum vitamin D levels are correlated with sleep disturbance in HD patients was examined.

## 2. Methods

### 2.1. Study Population

Patients undergoing maintenance HD with a frequency of three times per week were consecutively recruited at the HD center of the First Affiliated Hospital of Jiaxing University between 12 May 2014 and 20 June 2015. Eligibility criteria included: (i) Chinese ethnicity; (ii) aged over 18 years; (iii) receipt of maintenance HD therapy (single-pool Kt/V > 1.0) for at least 3 months; (iv) having the ability and willingness to give informed consent. Exclusion criteria were: (i) patients with severe visual or auditory impairment or cognitive dysfunction; (ii) patients with malignancy, autoimmune diseases or active infections; (iii) current treatment of immunosuppressants, immunomodulators or steroids; (iv) patients with a history of psychiatric disorders (clinical diagnosis or previous treatment); (v) concomitant chronic obstructive pulmonary disease and sleep apnea (clinical diagnosis or previous treatment); (vi) patients taking vitamin D supplementation or with osteoporosis. Meanwhile, 117 healthy volunteers without renal impairment, concomitant chronic obstructive pulmonary disease, sleep apnea, sleep complaints or a history of psychiatric disorders, were recruited from a health survey. Written informed consents were obtained from all participating subjects according to the principles of the Declaration of Helsinki (1989) and the study was approved by the Ethics Committee of the First Affiliated Hospital of Jiaxing University. The methods were carried out in accordance with the approved guidelines.

### 2.2. Clinical Variables

Demographic and clinical data including time on HD and dialysis shift were obtained through participant report and electronic medical records. Blood samples were collected according to a standard protocol between 8 a.m. and 10 a.m. each morning. Blood samples were obtained for all subjects and stored at −80 °C until measurement. All blood samples of the controls were collected at the end of August 2014. Serum 25-hydroxyvitamin D [25(OH)D] was chosen to measure vitamin D status for all subjects because of its widespread clinical application, standardized ranges and testing protocol. Serum 25(OH)D levels were test by using a competitive protein-binding assay at our hospital’s laboratory. The intra-assay coefficient of variation was 7%–10%. Serum 25(OH)D in HD patients was recorded and divided into four quartiles (≤26.0, 26.1–48.0, 48.1–84.0 and ≥84.1 nmol/L), as the raw 25(OH)D data were skewed. Blood pressure was measured at admission using an automated sphygmomanometer after at least 5 min of rest.

### 2.3. Measurement

Sleep quality was measured using the Pittsburgh Sleep Quality Index (PSQI) [20]. The self-administered questionnaire assesses individual’s sleep quality during the past month and contains 19 items which yield seven components, including subjective sleep quality, sleep latency, sleep duration, sleep efficiency, sleep disturbance, use of sleep medications, and daytime dysfunction. Each component is scored from 0 to 3, which yields a total PSQI score from 0 to 21, with high PSQI score indicating poor sleep quality. According to Buysse et al., subjects with a PSQI score ≥5 are considered as “poor sleepers”, while those with a score <5 are defined as “good sleepers”. The Chinese Version of PSQI has been used in Taiwan [21].

### 2.4. Statistical Analysis

Data were analyzed using SPSS for windows (SPSS Inc., Chicago, IL, USA) version 17. All continuous variables are expressed as the mean ± standard deviation (SD). The categorical variables were compared using the Pearson χ^2^ test. Student’s *t* test or one-way analysis of variance (ANOVA) was used for normally distributed variables, while the Mann-Whitney *U* test was performed for parametric variables with non-normal distributions. Post hoc tests were conducted to determine the difference between groups, followed by Fisher’s least significant difference (LSD) test. The association between serum 25(OH)D levels and sleep disturbance in HD patients was evaluated by logistic regression (Enter method) including all factors with *p* < 0.05 in the univariate analysis. The results were expressed as adjusted odds ratios (OR) with the corresponding 95% confidence intervals (CI). Level of statistical significance was defined as *p* < 0.05.

## 3. Results

### 3.1. Group Differences in Demographic and Clinical Information

A total of 141 HD patients (86 men, 55 women) with a mean age of 68 years were enrolled in the current study. The PSQI total score was 8.5 ± 4.3 and 88 patients (62.4%) were poor sleepers with PSQI global scores ≥5. The sub-scores were as follows: sleep quality, 1.2 ± 0.63; sleep onset latency, 1.4 ± 1.20; sleep duration, 1.54 ± 1.14; sleep efficacy, 0.29 ± 0.85; sleep disturbance 1.72 ± 0.79; daytime dysfunction, 2.23 ± 1.21. None of them received vitamin D supplementation and psychiatric drugs. There were no significant differences in mean age and gender (M/F) between HD patients and healthy volunteers. No between-season sampling differences were observed for the 25(OH)D levels in HD patients: spring (*n* = 17), 46.7 ± 30.7 nmol/L; summer (*n* = 60), 58.9 ± 37.5 nmol/L; fall (*n* = 51), 60.3 ± 41.8 nmol/L; and winter (*n* = 13), 44.2 ± 27.9 nmol/L (*p* = 0.40).

HD patients showed markedly lower serum 25(OH)D levels as compared to normal controls (56.6 ± 39.5 vs. 68.8 ± 19.9 nmol/L, *p* = 0.002). The serum 25(OH)D levels were significantly lower in poor sleepers than in good sleepers (39.1 ± 29.1 vs. 85.6 ± 37.4 nmol/L, *p* < 0.001). Furthermore, significant differences were observed between poor sleepers and good sleepers in 25(OH)D level quartiles of patients (*p* < 0.001). Indeed, the proportion of subjects in lower quartiles (≤26.0 and 26.1–48.0 nmol/L) was significantly higher in patients with sleep disturbance (both *p* < 0.001), while the proportion of subjects in higher quartiles (48.1–84.0 and ≥ 84.1 nmol/L) was significantly lower in patients with sleep disturbance (both *p* < 0.001) (Table 1).

When compared to good sleepers, the poor sleepers had spent a longer time on HD (2 (1–4) vs. 1 (1–2) years, *p* < 0.001), had a lower serum hemoglobin level (90.99 ± 18.30 vs. 109.96 ± 11.05 g/L, *p* < 0.001), a lower serum albumin level (32.54 ± 5.72 vs. 37.73 ± 3.63 g/L, *p* < 0.001), a lower serum calcium level (2.08 ± 0.21 vs. 2.24 ± 0.24 mmol/L, *p* < 0.001), a higher phosphate level (1.59 ± 0.57 vs. 1.56 ± 0.45 mmol/L, *p* = 0.024), and a lower HDL-C level (0.87(0.72–1.11) vs. 1.03(0.84–1.21) mmol/L, *p* = 0.004) (Table 1).

### 3.2. Independent Characteristics of Patients with Sleep Disturbance

With all HD patients taken as a whole, the presence of sleep disturbance taken as a dependent variable, and quartile 3 and quartile 4 taken as the references used for levels of serum 25(OH)D in the logistic analysis, 25(OH)D levels (≤48.0 nmol/L) were independently associated with the presence of sleep disturbance in HD patients (OR 9.897, 95% CI 3.356–29.187, *p* < 0.001). Moreover, lower levels of hemoglobin, lower levels of albumin and higher levels of phosphorus were significantly associated with the presence of sleep disturbance in HD patients (OR 0.954, 95% CI 0.921–0.988, *p* = 0.009; OR 0.867, 95% CI 0.760–0.989, *p* = 0.034 and OR 3.423, 95% CI 1.061–11.044, *p* = 0.039, respectively) (Table 2).

## 4. Discussion

To the best of our knowledge, this is the first study to analyze the association between serum levels of vitamin D and sleep quality in HD patients. We found that low serum levels of vitamin D were significantly associated with sleep disturbance in HD patients, which is similar to the findings of previous studies in elderly adults and patients with SLE [12,13,14]. Our finding might have important implications in providing novel proposals for the prevention and treatment of sleep disturbance in HD patients. 

In the present study, we found that 62.4% of HD patients had sleep disturbance, which is consistent with the findings of recent research [4,22]. Currently, despite many available documents, it remains difficult to determine the true prevalence of sleep disturbance in HD patients, possibly due to these differences in study designs, the time of sleep evaluation, the source of patient recruitment, and race/ethnicity [23]. Our results also demonstrated that lower levels of hemoglobin, lower levels of albumin and higher levels of phosphorus were risk factors for the development of sleep disturbance in HD patients, which broadly agrees with the results of earlier studies [4,24,25]. 

We found that serum levels of vitamin D were significantly lower in poor sleepers than in good sleepers. Sleep disturbance is associated with increased food intake and obesity which is an important risk factor for vitamin D deficiency [26,27]. Nevertheless, there was no intergroup difference in BMI in our HD cohort. Since serum levels of vitamin D are directly related to sunlight exposure in humans [8], low vitamin D levels of the poor sleepers could result from decreased exposure to sunlight.

Importantly, we found that serum vitamin D levels were independently associated with the presence of sleep disturbance in HD patients. As mentioned earlier, a growing body of studies has reported low vitamin D levels with sleep disturbance in non-HD subjects [12,13,14]. Moreover, several uncontrolled clinical trials have demonstrated the positive effects of vitamin D supplementation on sleep quality in non-HD subjects [15,16]. The exact mechanisms by which vitamin D could affect sleep are unclear. In animal studies, vitamin D receptors have been found in specific regions of the central nervous system, some of which regulate sleep, including the anterior and posterior hypothalamus, the raphe nuclei, the midbrain central gray, and the nucleus reticularis pontis caudalis and oralis [9,10,11,28], which suggests vitamin D may play a role in individuals’ sleep. Another possible explanation is the effect of vitamin D on the immune system. Vitamin D plays a key role in modulating the secretion of inflammatory cytokines, such as tumor necrosis factor-α (TNF-α), interleukin-6 (IL-6), and interleukin-1β (IL-1β) [29,30,31]. A growing body of evidence suggests the involvement of IL-1β, TNF-α, and other inflammatory cytokines in sleep regulation [32,33]. Plasma levels of IL-1, IL-6 and TNF-α are increased in maintenance HD patients [34,35], which correlates with a poor outcome in these patients [36,37]. Vitamin D deficiency is extremely common in HD patients and is an independent predictor of disease progression and mortality in these patients [17,18,19]. Therefore, these results mentioned above suggest that vitamin D might play an important role in sleep disturbance in HD patients.

Some limitations of the present study should be noted. First, the association between vitamin D levels and sleep traits has been reported to vary by race/ethnicity [23]. Subjects in our sample were recruited from the Han Chinese population. Therefore, our results may not be readily generalized to other populations. Second, the effects of other variables on vitamin D levels, such as dietary intake and lifestyle changes, were not considered in the current study. Finally, we did not perform an objective sleep assessment such as a polysomnography to prove our findings. Further studies with a polysomnogram or other objective measures are needed. However, the correlation between the PSQI and polysomnography has been shown to be significant in certain domains [38].

## 5. Conclusions

In summary, despite of these limitations, our study demonstrates an important association between serum levels of vitamin D and sleep disturbance in patients undergoing maintenance HD. However, the finding would have to be confirmed in future experimental and clinical studies.

## Figures and Tables

**Table 1:** Demographic, clinical and laboratory characteristics of the sample.

Variables	Poor Sleepers (PSQI ≥ 5, *n* = 88)	Good Sleepers (PSQI < 5, *n* = 53)	Normal Controls (*n* = 117)
Gender (M/F)	52/36	34/19	68/49
Age (years)	59.7 ± 15.3	62.8 ± 12.5	60.1 ± 14.0
BMI (kg/m^2^)	22.6 ± 3.4	21.7 ± 2.7	22.8 ± 2.9
Education level 1/2 ^a^	21 (23.9)	16 (30.2)	
Widowed	11 (11.7)	3 (5.7)	
Current smoking	18 (20.5)	9 (17.0)	
Current drinking	11 (12.5)	6 (11.3)	
Cause of renal failure			
Diabetes	28 (31.8)	15 (28.3)	
Hypertension	28 (31.8)	11 (20.8)	
Glomerulonephritis	16 (18.2)	14 (26.4)	
Other	16 (18.2)	13 (24.5)	
Time on HD (years)	2 (1–4) ^b^	1 (1–2)	
Dialysis shift (morning/evening)	54/34	23/20	
SBP (mmHg)	148.6 ± 27.3	141.6 ± 32.7	
DBP (mmHg)	76.9 ± 15.0	76.7 ± 15.0	
Hemoglobin (g/L)	91.0 ± 18.3 ^b^	111.0 ± 11.1	
Albumin (g/L)	32.5 ± 5.7 ^b^	37.7 ± 3.6	
Fasting glucose (mmol/L)	4.7 (4.0–5.9)	4.5 (3.9–6.8)	
Creatinine (FV)	600.6 ± 262.1	649.7 ± 255.1	
Uric acid (μmol/L)	358.3 ± 166.8	332.4 ± 121.0	
Calcium (mmol/L)	2.08 ± 0.21 ^b^	2.24 ± 0.24	
Phosphate (mmol/L)	1.59 ± 0.57 ^c^	1.56 ± 0.45	
iPTH (pg/mL)	239.9 (124.9–431.0)	211.9 (90.0–314.4)	
Triglyceride (mmol/L)	1.55 (1.17–2.25)	1.60 (1.11–2.65)	
Total cholesterol (mmol/L)	4.29 (3.57–5.11)	4.36 (3.70–5.60)	
LDL-C (mmol/L)	2.58 (1.98–3.09)	2.42 (1.94–3.07)	
HDL-C (mmol/L)	0.87 (0.72–1.11) ^c^	1.03 (0.84–1.21)	
25(OH)D ^d^			
Quartile 1	36 (40.9) ^b^	3 (5.7)	
Quartile 2	28 (31.8) ^b^	4 (7.5)	
Quartile 3	12 (13.6) ^b^	23 (43.4)	
Quartile 4	12 (13.6) ^b^	23 (43.4)	
25(OH)D (nmol/L)	39.1 ± 29.1 ^b,e^	85.6 ± 37.4 ^e^	68.8 ± 19.9

Data are expressed as number (percentage) or means (±SD) or medians (IQR). Abbreviations: PSQI, Pittsburgh Sleep Quality Index; BMI, body mass index; HD, hemodialysis; SBP, systolic blood pressure; DBP, diastolic blood pressure; iPTH, intact parathyroid hormone; LDL-C, low-density lipoprotein cholesterol; HDL-C, high-density lipoprotein cholesterol; 25(OH)D, 25-hydroxyvitamin D. ^a^ 1 Bachelor or above, 2 elementary school, Junior high school, senior high school; ^b^ *p* < 0.001 compared with good sleepers; ^c^ *p* < 0.05 compared with good sleepers; ^d^ *p* < 0.001 between poor sleepers and good sleepers in 25(OH)D level quartiles of patients; ^e^ *p* < 0.001 compared with normal controls.

**Table 2:** Characteristics associated with sleep disturbance in HD patients ^a^.

Variables	OR (95% CI)	*p* Value
25(OH)D ^b^	9.897 (3.356–29.187)	<0.001
Time on HD		0.687
Hemoglobin	0.954 (0.921–0.988)	0.009
Albumin	0.867 (0.760–0.989)	0.034
Calcium		0.306
Phosphate	3.423 (1.061–11.044)	0.039
HDL-C		0.715

25(OH)D, 25-hydroxyvitamin D; HD, hemodialysis; OR, odds ratio; CI, confidence interval; HDL-C, high-density lipoprotein cholesterol. ^a^ Contains only the variables which were significant (*p <* 0.05) in the multivariable model; ^b^ Quartile 1 and quartile 2.
